# Dietary Polyphenols in Non‐Communicable Chronic Diseases: Neuro–Enteric Mechanisms, Multi‐Omics Biomarkers and Translational Opportunities

**DOI:** 10.1002/fsn3.71856

**Published:** 2026-05-01

**Authors:** Adnan Akif, Jannatul Wahid Munami, Rajib Das, Nusrat Jahan Shawon

**Affiliations:** ^1^ Department of Physiology & Cell Biology School of Medicine, University of Nevada Reno Nevada USA; ^2^ Department of Pharmacy Primeasia University Dhaka Bangladesh; ^3^ Department of Pharmacy Jahangirnagar University Dhaka Bangladesh; ^4^ Department of Pharmacy Independent University Dhaka Bangladesh

**Keywords:** cognition, endothelial function, gut–brain axis, microbiome, polyphenols, randomized trials

## Abstract

Polyphenols from plant foods (tea, cocoa, berries, grapes, and extra‐virgin olive oil) modulate oxidative stress, inflammation, vascular function, and the gut microbiome—axes central to non‐communicable chronic diseases (NCCDs) that involve the brain and enteric nervous system (ENS). Recent randomized trials and longitudinal studies report modest but reproducible benefits on cognitive domains and vascular/endothelial function with berry/grape extracts, matcha/green tea, and high‐polyphenol extra‐virgin olive oil; effects appear stronger in older adults or those with metabolic risk. Complementary evidence in irritable bowel syndrome (IBS)—a prototypical gut–brain disorder—suggests polyphenol‐based combinations (often with probiotics/fiber) can improve quality of life and inflammatory markers, supporting enteric–central crosstalk. Emerging genetics (Mendelian randomization) and multi‐omics readouts strengthen causal inferences for tea polyphenols in neurodegeneration‐adjacent outcomes and outline mechanistic mediators (endothelial/BBB function, cytokine tone, microbiome‐derived metabolites). Key gaps remain: heterogeneous formulations/doses, limited head‐to‐head trials, sparse target engagement biomarkers, and uncertain durability after discontinuation. We synthesize clinical and mechanistic advances, propose a standardized biomarker set (neurocognitive, endothelial, immune, and microbiome‐metabolome), and outline designs for mechanism‐anchored RCTs that integrate ENS endpoints with brain outcomes to translate associative signals into precision nutrition strategies for NCCDs.

## Graphical abstract

This illustration highlights how dietary polyphenols from foods like berries, grapes, and green tea travel through the gut, where microbiota transform them into bioactive metabolites. These metabolites pass into the bloodstream, strengthen the gut barrier, and ultimately reach the brain, supporting neuroprotection through antioxidative and anti‐inflammatory effects.

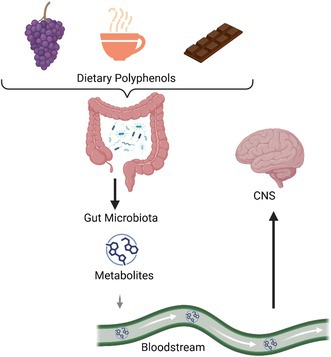

**Abbreviations**
AMP: Adenosine monophosphate (part of AMP‐activated protein kinase, AMPK)
BBB: Blood–brain barrier
CNS: Central nervous system
COMT: Catechol‐O‐methyltransferase (gene affecting polyphenol metabolism
COSMOS: Cocoa Supplement and Multivitamin Outcomes Study
CRP (hs‐CRP): (High‐sensitivity) C‐reactive protein
CSF: Cerebrospinal fluid
EGCG: Epigallocatechin gallate (a catechin in green tea)
ENB‐2 (ENB): Esame Neuropsicologico Breve 2 (neuropsychological test battery)
ENS: Enteric nervous system
EVOO: Extra‐virgin olive oil
FMD: Flow‐mediated dilation (measure of endothelial function)
GAD‐7 (GAD): Generalized Anxiety Disorder‐7 questionnaire
GM‐CSF (GM): Granulocyte‐macrophage colony‐stimulating factor
HDL: High‐density lipoprotein
HOMA‐IR (HOMA): Homeostasis model assessment of insulin resistance
IBS: Irritable bowel syndrome
IL: Interleukin (e.g., IL‐6, IL‐8)
IR: Insulin resistance
LDL: Low‐density lipoprotein
LPS: Lipopolysaccharide (endotoxin)
MCI: Mild cognitive impairment
MMSE: Mini‐Mental State Examination
NADPH: Nicotinamide adenine dinucleotide phosphate (cofactor in oxidative pathways)
NCCD (NCCDs): Non‐communicable chronic disease(s)
PHGG: Partially hydrolyzed guar gum
PHQ‐9 (PHQ): Patient Health Questionnaire‐9 (depression assessment)
PSQI: Pittsburgh Sleep Quality Index
PWV: Pulse wave velocity
RBANS: Repeatable Battery for the Assessment of Neuropsychological Status
RCT: Randomized controlled trial
SCFA: Short‐chain fatty acid
SD: Standard deviation
TNF (TNF‐α): Tumor necrosis factor (alpha)

## 1 Introduction

Non‐communicable chronic diseases account for the majority of morbidity and mortality worldwide. NCCDs share pathobiological drivers—chronic oxidative stress, low‐grade inflammation, and vascular dysfunction—that affect both the central nervous system (CNS) and the enteric nervous system (ENS). Oxidative stress and inflammation have been recognized as central mechanisms in cardiovascular, diabetic, and neurodegenerative diseases (Nediani et al. 2024). Gut–brain disorders such as irritable bowel syndrome (IBS) also display systemic inflammation, altered gut permeability, and heightened stress signaling. Because the gut and brain communicate bidirectionally via neural, immune, and endocrine pathways, interventions that modulate both systems may provide broad benefits.

Polyphenols possess antioxidant and anti‐inflammatory properties. They scavenge reactive oxygen species, chelate metal ions, and up‐regulate endogenous antioxidant enzymes, thereby dampening oxidative stress (Crescente and Moccia 2024). Flavonoids such as quercetin and kaempferol reduce pro‐inflammatory cytokines (IL‐6, TNF‐α) and inhibit vascular calcification (Crescente and Moccia 2024). Polyphenols also modulate endothelial function by enhancing nitric oxide synthase activity, improving flow‐mediated dilation (FMD), and reducing oxidative modifications of LDL. These actions are relevant for cognitive aging because cerebral perfusion and blood–brain barrier integrity rely on vascular health (Barona et al. 2012; Leikert et al. 2002). In the gut, polyphenols act as prebiotics: they promote the growth of beneficial taxa (Bifidobacterium, Lactobacillus) and suppress pathogens (Rodríguez‐Daza et al. 2021). Bifidobacteria metabolize polyphenols into short‐chain fatty acids (SCFAs) and urolithins, which have anti‐inflammatory and neuroprotective effects (Sarubbo et al. 2023). Polyphenols strengthen the intestinal barrier and reduce endotoxemia; cocoa bean shell extracts protected against oxysterol‐induced intestinal damage and improved gut microbiota composition in preclinical models (Alia et al. 2024). Through these mechanisms, polyphenols may influence ENS signaling and systemic inflammation, ultimately affecting brain function. Upon ingestion, polyphenols undergo extensive metabolism. In the small intestine, only simple phenolics and flavonoid aglycones are absorbed; most complex glycosides and polymeric proanthocyanidins reach the colon, where the gut microbiota cleaves sugar moieties and catabolizes ring structures (Duda‐Chodak et al. 2015). This produces low‐molecular‐weight metabolites, including phenyl‐γ‐valerolactones, phenolic acids and urolithins (from ellagitannins). These metabolites, rather than the parent compounds, are often the bioactive forms.

While many epidemiological studies correlate polyphenol‐rich diets (e.g., Mediterranean diet) with reduced NCCD risk, causality is uncertain due to confounding and measurement error. Heterogeneity in polyphenol content between foods, variation in bioavailability and inter‐individual differences in gut microbiome composition complicate interpretation. Controlled trials provide more robust evidence but vary widely in doses, formulations, populations and endpoints. Few studies include both CNS and ENS outcomes, and biomarkers of target engagement (e.g., plasma metabolites, FMD, cytokines) are inconsistently reported. The gut and brain are engaged in constant, bidirectional communication through a network of neural (e.g., the vagus nerve), immune (e.g., cytokines), and endocrine pathways, heavily influenced by metabolites produced by the gut microbiota. Interventions capable of beneficially modulating this axis may therefore confer broad, multi‐system health benefits. Dietary polyphenols, a vast and structurally diverse class of plant‐derived secondary metabolites, have emerged as a leading candidate for such an intervention, offering a plausible, food‐based approach to mitigating the shared drivers of NCCDs.

### 1.1 Scope and Approach

This narrative review synthesizes human evidence on dietary polyphenols and related microbiota‐derived metabolites in NCCDs at the neuro–enteric interface, emphasizing cognitive aging, vascular/endothelial dysfunction, and disorders of gut–brain interaction, with irritable bowel syndrome (IBS) used as a prototypical model. Inclusion is limited to adult human studies of polyphenol‐containing foods, extracts, or standardized supplements reporting outcomes in cognition, vascular function, gastrointestinal symptom burden, and systemic inflammatory/oxidative‐stress biomarkers; mechanistic multi‐omics is used only to contextualize pathways and candidate biomarkers. We exclude disease areas not centrally covered here (e.g., cancer and chronic respiratory disease) and do not attempt to provide clinical practice recommendations or guideline‐grade policy advice; accordingly, this article is a narrative synthesis rather than a systematic review. Randomized controlled trials are prioritized for causal inference on intervention effects, observational cohorts are used mainly for exposure–outcome associations, and Mendelian randomization is used as complementary causal triangulation subject to instrumental‐variable assumptions and sensitivity analyses.

## 2 Dietary Polyphenols: Chemical and Metabolic Overview

This section asks which polyphenol classes, food sources, and biotransformation steps are most relevant for interpreting downstream human outcomes. It provides the chemical and metabolic foundation needed to understand why the clinical responses discussed next vary by formulation, host microbiome, and absorbed metabolite profile.

### 2.1 Classification and Dietary Sources

**FIGURE 1:**
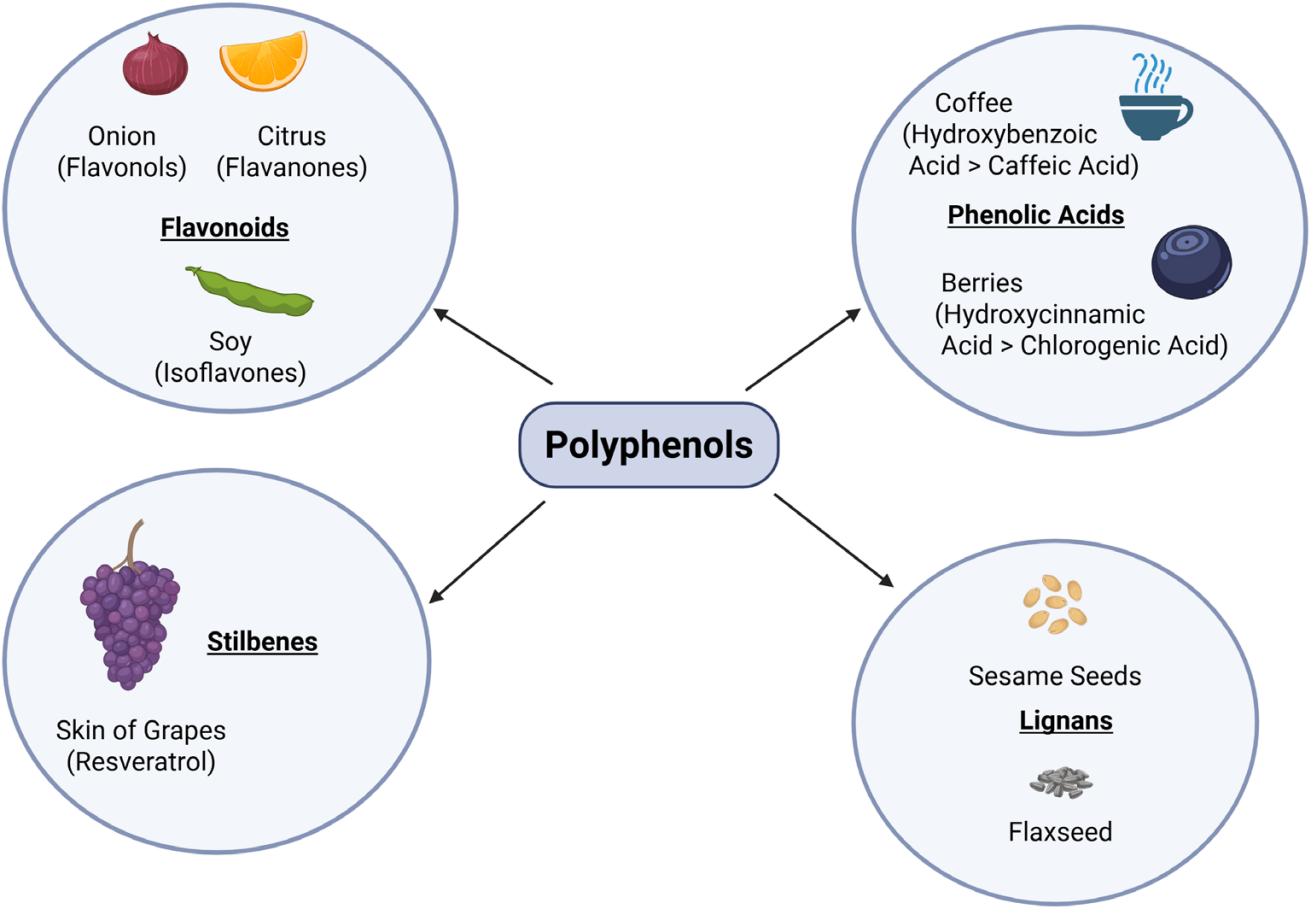
Classification and primary dietary sources of polyphenols. This schematic categorizes polyphenols into four main subclasses: flavonoids, phenolic acids, stilbenes, and lignans.

Polyphenols are broadly categorized into four main subclasses, each with unique chemical properties and dietary distributions (Shown in Figure 1): (a) Flavonoids: This is the largest and most studied subgroup, further divided into several families, including flavanols (e.g., quercetin in onions, kaempferol), flavones (e.g., luteolin), flavanols (e.g., catechins like epigallocatechin gallate [EGCG] in green tea), flavanones (e.g., naringenin in citrus), anthocyanins (responsible for the red, blue, and purple pigments in berries and grapes), and isoflavones (e.g., genistein in soy) (Rodríguez Galdón et al. 2008; Singh et al. 2011). (b) Phenolic Acids: These compounds include hydroxybenzoic and hydroxycinnamic acids. Notable examples are caffeic acid and chlorogenic acid, which are particularly abundant in coffee, berries, and whole grains (Mattila et al. 2006). (c) Stilbenes: This smaller class is best known for resveratrol, a compound famously found in the skin of grapes and in red wine (Brâkenhielm et al. 2001). (d) Lignans: These are found in high concentrations in seeds, particularly flaxseed and sesame seeds, with secoisolariciresinol being a primary example (Gerstenmeyer et al. 2013). The richest dietary sources of polyphenols are overwhelmingly plant‐based and include fruits (especially berries, grapes, and pomegranates), vegetables (onions, artichokes), beverages (green tea, coffee, cocoa, red wine), and extra‐virgin olive oil (EVOO).

### 2.2 Metabolism and Bioavailability: The Central Role of the Microbiome

The biological activity of dietary polyphenols is profoundly influenced by their metabolic fate following ingestion. A common misconception is that these compounds are absorbed directly in their parent form. The bioavailability of most polyphenols is low. Only a small fraction of simple phenolics and flavonoid aglycones (the non‐sugar‐bound form) are absorbed in the small intestine (Hollman 2004). Many ingested polyphenols, particularly complex polymers like proanthocyanidins and glycosides (sugar‐bound forms), pass undigested into the colon (Renard et al. 2017).

It is in the colon that the gut microbiota plays a pivotal role, acting as a “metabolic engine” to transform these complex molecules into smaller, more bioavailable, and often more bioactive compounds. The diverse enzymatic machinery of colonic bacteria cleaves sugar moieties and catabolizes the core ring structures of polyphenols, producing a suite of low‐molecular‐weight metabolites. For example, ellagitannins, found in pomegranates, walnuts, and berries, are converted by specific gut microbes into a class of compounds known as urolithins (Landete 2011; Selma et al. 2018). This biotransformation is essential; the health effects attributed to many polyphenol‐rich foods are likely mediated not by the parent compounds themselves, but by the unique metabolites produced by an individual's gut microbiome. This dependency on microbial metabolism also explains the significant inter‐individual variability observed in response to nutritional interventions, as the capacity to produce specific metabolites is contingent on possessing the necessary microbial species.

## 3 Clinical Signals: CNS Endpoints

Having outlined the major compound classes and their metabolism, the next question is whether these mechanisms translate into measurable human brain‐related outcomes. This section therefore reviews intervention studies on cognition and related CNS endpoints before the discussion expands to enteric and gut–brain outcomes.

### 3.1 Large‐Scale Trials: Lessons From the Cocoa Supplement and Multivitamin Outcomes Study (COSMOS)

The Cocoa Supplement and Multivitamin Outcomes Study (COSMOS) is the largest randomized trial evaluating cocoa flavanols and multivitamin–mineral supplementation on cognition. In the *COSMOS‐Mind* substudy (*n*≈21,442), participants aged ≥ 60 years were randomized to cocoa extract (500 mg/day cocoa flavanols) and/or multivitamin–mineral supplementation for 3 years. The cocoa extract did not improve global cognition (mean difference 0.03 SD units) or memory (Baker et al. 2023). In contrast, multivitamin–mineral supplementation produced a small but significant improvement in global cognition (mean difference 0.07 SD; *p*≈0.007) and benefited episodic memory and executive function, particularly among participants with cardiovascular disease (Baker et al. 2023). A subsequent meta‐analysis of COSMOS substudies with in‐person assessments reported that multivitamin supplementation improved global cognition by 0.06 SD and episodic memory by 0.12 SD, equivalent to offsetting 2 years of age‐related cognitive decline (Vyas et al. 2024). This dichotomy within a single trial provides a crucial lesson: the “food matrix” and background nutritional status matter. The failure of a high‐dose, isolated flavanol extract, juxtaposed with the success of a broad‐spectrum micronutrient supplement, suggests that addressing baseline nutritional insufficiencies may be a prerequisite for other dietary interventions to confer a benefit. It points away from a single “magic bullet” compound and toward the importance of overall dietary quality and nutrient sufficiency.

### 3.2 Tea‐Derived Polyphenols: Matcha, Green Tea, and Insights

Green tea contains catechins (epigallocatechin gallate, EGCG), L‐theanine, and caffeine. In a 12‐month, double‐blind randomized controlled trial of 60 older adults with mild cognitive decline, daily matcha powder (2 g) was compared with placebo. The matcha group showed significant improvement in social acuity (emotion perception) (−1.39 points vs. placebo, *p* = 0.028) and a trend toward improved sleep quality, but no significant difference in overall cognitive scores (Uchida et al. 2024). Improvements in social cognition and sleep may reflect the combined effects of catechins and L‐theanine on GABAergic signaling and stress reduction. Genetic studies provide complementary evidence; a Mendelian‐randomization analysis using genome‐wide association data reported that genetically higher green‐tea intake was associated with a 13% reduction in progression to dementia among individuals with Parkinson's disease and nominally associated with slower progression to depression (Li et al. 2022). These findings support a causal role for tea‐related biology in neurodegeneration, although the specific metabolites mediating the effects remain to be identified.

### 3.3 Grape and Blueberry Extracts

Extracts and powders derived from grapes and blueberries, rich in anthocyanins and proanthocyanidins, have been the focus of several promising trials.

#### 3.3.1 Grape/Blueberry Extract

In a six‐month RCT of 142 individuals with mild cognitive impairment (MCI), supplementation with 600 mg/day of a standardized grape and blueberry extract (Memophenol) led to significant improvements in the speed of information processing (*p* = 0.020) and visuospatial learning (*p* = 0.012) compared to placebo (Lopresti et al. 2023). No serious adverse effects occurred. This finding is particularly relevant as it demonstrates a benefit in a population already experiencing cognitive deficits, suggesting a restorative rather than purely preventative effect.

#### 3.3.2 Standardized Grape Extract

In a 12‐week double‐blind trial of 88 healthy older adults, daily ingestion of 250 mg of a standardized *Vitis vinifera* extract (Cognigrape) led to rapid and sustained cognitive improvements. The Mini‐Mental State Evaluation (MMSE) score increased by 4.6% and 8.9% after 14 and 84 days, changes that were significantly greater than placebo (Amone et al. 2024). The Repeatable Battery for the Assessment of Neuropsychological Status (RBANS) score improved by 9.5% and 14.4% after 28 and 84 days, respectively, with significant gains across immediate memory, visuospatial construction, language, and attention (Amone et al. 2024). These benefits suggest that grape polyphenols, which contain anthocyanins, proanthocyanidins, and resveratrol derivatives, can enhance multiple cognitive domains even in cognitively normal adults.

#### 3.3.3 Wild Blueberry Trial

The *Wild Blueberry* trial enrolled 61 healthy older adults in a double‐blind crossover design. Daily intake of 26 g freeze‐dried wild blueberry powder (providing ≈302 mg anthocyanins) for 12 weeks significantly improved flow‐mediated dilation (FMD) and enhanced immediate word recall and task switching compared with placebo (Wood et al. 2023). Twenty‐four‐hour systolic blood pressure decreased in the blueberry group (−5 mmHg) and urinary total polyphenols increased, indicating good absorption and compliance (Wood et al. 2023). These findings support vascular mechanisms for cognitive benefits; improved FMD correlated with enhanced episodic memory.

### 3.4 The Mediterranean Keystone: High‐Polyphenol Extra‐Virgin Olive Oil

Extra‐virgin olive oil (EVOO) is a cornerstone of the Mediterranean diet and rich in hydroxytyrosol and oleuropein. In the *OLIVAUS* cross‐over trial, 50 adults consumed either high‐polyphenol EVOO (320 mg/kg) or low‐polyphenol EVOO (86 mg/kg) for 3 weeks each. High‐polyphenol EVOO consumption decreased plasma oxidized LDL and increased total antioxidant capacity, particularly among participants with abdominal obesity (Sarapis et al. 2022). A more recent randomized trial of hyperlipidemic patients compared low‐dose high‐phenolic EVOO (25 mL/day, 500 mg/kg phenolics) with high‐dose low‐phenolic EVOO (50 mL/day, 250 mg/kg). The high‐phenolic low‐dose group achieved greater reductions in total cholesterol, LDL, and lipoprotein (a) and increased HDL (Kourek et al. 2025). Although cognitive outcomes were not measured, improved lipid profiles and antioxidant status may indirectly benefit brain and vascular health.

Other polyphenols with clinically relevant but still mixed translational signals deserve brief mention. Curcumin is especially notable because formulation‐dependent bioavailability appears to be a major determinant of efficacy. In an 18‐month double‐blind placebo‐controlled trial in non‐demented middle‐aged and older adults, a bioavailable curcumin preparation improved memory and attention and was associated with lower amyloid and tau signal in selected brain regions, whereas earlier trials using other formulations reported more limited or inconsistent benefit (Small et al. 2018). Contemporary syntheses emphasize that microbiota‐derived phenolic metabolites are biologically plausible mediators of neurocognitive effects, but they also note that direct human evidence remains limited by small trials, variable biomarker strategies, and incomplete characterization of blood–brain barrier penetration and host metabotype (Domínguez‐López et al. 2025). To provide a structured and verifiable overview of the currently cited clinical evidence, we have added a consolidated summary table of key randomized trials, including study population, intervention characteristics, follow‐up duration, major endpoints, principal outcomes, effect sizes/statistical significance where reported, and available adherence information (Table 1).

**TABLE 1:** Summary of key clinical trials of dietary polyphenol interventions, including study population, intervention and dose, duration, principal endpoints, main outcomes, effect sizes/statistical significance and adherence events.

Study/trial	Population (*n*)	Intervention and dose	Follow‐up/design	Primary/key endpoints	Main outcomes	Effect size/statistical significance	Adherence
COSMOS‐Mind (Baker et al. 2023)	Older adults aged ≥ 60 years (*n*≈21,442)	Cocoa extract, 500 mg/day cocoa flavanols; randomized alongside multivitamin‐mineral arm	3 years, randomized trial	Global cognition, memory, episodic memory, and executive function	Cocoa extract did not improve global cognition or memory. In contrast, multivitamin‐mineral supplementation produced a small but significant benefit in global cognition and also benefited episodic memory and executive function, especially in participants with cardiovascular disease	Cocoa extract: global cognition mean difference 0.03 SD units; no significant benefit reported. Multivitamin‐mineral: global cognition mean difference 0.07 SD, p≈0.007	No result reported
Matcha green tea trial (Uchida et al. 2024)	Older adults with mild cognitive decline (*n* = 60)	Matcha powder 2 g/day vs. placebo	12 months, double‐blind randomized controlled trial	Overall cognitive scores, social acuity (emotion perception), and sleep quality	Matcha significantly improved social acuity and showed a trend toward improved sleep quality, but no significant difference in overall cognitive scores	Social acuity: −1.39 points vs. placebo, *p* = 0.028; overall cognition not significant	No result reported
Memophenol grape/blueberry extract trial (Lopresti et al. 2023)	Older adults with mild cognitive impairment (*n* = 142)	Standardized grape and blueberry extract, 600 mg/day	6 months, randomized, double‐blind, placebo‐controlled trial	Cognitive function, especially processing speed and visuospatial learning	Significant improvements were reported in speed of information processing and visuospatial learning compared with placebo; the manuscript notes that most other cognitive and self‐report outcomes were not significantly different	Processing speed: *p* = 0.020; visuospatial learning: *p* = 0.012	No result reported.
Standardized grape extract trial (Amone et al. 2024)	Healthy older adults (*n* = 88)	Standardized *Vitis vinifera* extract, 250 mg/day	12 weeks, double‐blind trial	MMSE, RBANS, immediate memory, visuospatial construction, language, attention	Rapid and sustained improvements in global and domain‐specific cognition compared with placebo	MMSE increased 4.6% after 14 days and 8.9% after 84 days; RBANS improved 9.5% after 28 days and 14.4% after 84 days; changes reported as significantly greater than placebo	No result reported
Wild blueberry powder trial (Wood et al. 2023)	Healthy older adults (*n* = 61)	Freeze‐dried wild blueberry powder, 26 g/day (providing≈302 mg anthocyanins)	12 weeks, double‐blind crossover design	Flow‐mediated dilation (FMD), immediate word recall, task switching, 24‐h systolic blood pressure, urinary total polyphenols	Blueberry intervention significantly improved FMD, immediate word recall, and task switching; 24‐h systolic blood pressure decreased; urinary total polyphenols increased, supporting exposure/compliance	24‐h systolic BP: −5 mmHg; significant improvements reported for FMD and selected cognitive endpoints	Urinary total polyphenols increased, indicating good absorption and compliance
OLIVAUS high‐polyphenol EVOO trial (Sarapis et al. 2022)	Adults (*n* = 50)	High‐polyphenol EVOO (320 mg/kg) vs. low‐polyphenol EVOO (86 mg/kg)	3 weeks each, double‐blind randomized controlled crossover study	Plasma oxidized LDL, total antioxidant capacity	High‐polyphenol EVOO decreased plasma oxidized LDL and increased total antioxidant capacity, particularly in participants with abdominal obesity; the article also notes no significant differences in the total sample for some outcomes, with effects more pronounced in higher‐risk strata	Direction of effect reported; exact *p*‐values/effect sizes not provided in current manuscript summary	No result reported
High‐phenolic EVOO in hyperlipidemia (Kourek et al. 2025)	Patients with hyperlipidemia (sample size not stated)	Low‐dose high‐phenolic EVOO (25 mL/day, 500 mg/kg phenolics) vs. high‐dose low‐phenolic EVOO (50 mL/day, 250 mg/kg)	Randomized clinical trial; duration not reported.	Total cholesterol, LDL, lipoprotein (a), HDL	The high‐phenolic low‐dose EVOO group achieved greater reductions in total cholesterol, LDL, and lipoprotein (a) and increased HDL. Cognitive outcomes were not measured	Exact effect sizes and *p*‐values not reported	No result reported

Abbreviations: COSMOS, Coocoa Supplement and Multivitamin Outcomes Study; EVOO, extra‐virgin olive oil; FMD, flow‐mediated dilation; HDL, high‐density lipoprotein; LDL, low‐density lipoprotein; MCI, mild cognitive impairment; MMSE, Mini‐Mental State Examination; RBANS, Repeatable Battery for the Assessment of Neuropsychological Status; RCT, randomized controlled trial; SD, standard deviation.

## 4 Clinical Signals: Enteric and Gut‐Brain Axis Endpoints

Because polyphenol actions are unlikely to be restricted to the brain alone, this section asks whether human studies also detect benefits at the level of gut symptoms, inflammatory tone, and microbiota‐linked gut–brain signaling. These enteric findings provide an essential bridge to the integrated mechanistic framework developed in the following section. Recent trials demonstrate direct effects on gut health and systemic inflammation in the context of gut–brain disorders.

### 4.1 Synbiotic Interventions in Irritable Bowel Syndrome: A Model for Gut‐Brain Modulation

IBS is characterized by abdominal pain, altered bowel habits, and psychological comorbidities. In 2025, Wierzbicka and colleagues conducted a randomized, double‐blind, placebo‐controlled trial evaluating a synbiotic blend containing polyphenol‐rich extracts (blueberry, blackcurrant, pomegranate), probiotics ( *Lactobacillus plantarum* and *Bifidobacterium longum* ), and partially hydrolyzed guar gum (PHGG) (Wierzbicka et al. 2025). Forty‐eight adults with IBS received the synbiotic or placebo for 8 weeks. The results demonstrated significant, multi‐level benefits. Clinically, the synbiotic group experienced a marked improvement in quality of life, with significant reductions in scores for dysphoria and bowel pattern disruption. At the systemic level, the intervention led to a significant decrease in serum concentrations of several pro‐inflammatory cytokines, including Interleukin‐8 (IL‐8), Tumor Necrosis Factor (TNF), and Granulocyte‐macrophage colony‐stimulating factor (GM‐CSF). Finally, analysis of stool samples confirmed target engagement within the gut: concentrations of beneficial short‐chain fatty acids (SCFAs)—acetate, propionate, and butyrate—were significantly increased, indicating robust microbial fermentation of the prebiotic fiber and polyphenols (Wierzbicka et al. 2025). This study provides a powerful proof‐of‐concept that combining polyphenols with pre‐ and probiotics can simultaneously ameliorate enteric symptoms and quell the systemic inflammation that links gut dysfunction to broader health outcomes.

## 5 Mechanistic Mediators Across the CNS–ENS

The central question in this section is which biological mediators plausibly connect the clinical signals described above across the brain, vasculature, immune system, microbiome, and enteric nervous system. Clarifying these pathways is necessary before proposing the biomarker and trial‐design priorities outlined in the future‐research framework. The gut and brain communicate bidirectionally via neural pathways (vagus nerve), cytokines, and microbial metabolites. Polyphenols can influence this axis by modulating gut microbiota composition and producing metabolites that cross the BBB. The IBS trial described above demonstrated that polyphenol‐probiotic‐fiber combinations decreased pro‐inflammatory cytokines and increased SCFAs, which are known to cross the BBB and support microglial function. Similar improvements in sleep quality were observed in the matcha trial (Uchida et al. 2024), suggesting that gut‐mediated anti‐inflammatory effects could translate to better sleep and mood. Observational studies link dietary polyphenols to reduced risk of depression and anxiety, and the Mediterranean diet—rich in polyphenols and fiber—has been shown to increase microbial diversity, elevate beneficial bacteria such as Bifidobacteria and *Faecalibacterium*, and reduce systemic inflammation (Lin et al. 2021; Merra et al. 2021; Qiu et al. 2013).

### 5.1 Vascular and Endothelial Pathways: A Primary Bridge Between Gut and Brain

Many cognitive benefits of polyphenols may be mediated by improvements in vascular and endothelial function. In the wild blueberry trial, increased flow‐mediated dilation (FMD) correlated with improved memory and executive function (Wood et al. 2023). High‐polyphenol EVOO reduced oxidized LDL and improved lipid profiles (Kourek et al. 2025; Sarapis et al. 2022), potentially enhancing cerebral perfusion. Polyphenols increase nitric oxide bioavailability, inhibit NADPH oxidase, and modulate endothelial gene expression. Vascular improvements also affect the ENS; increased gut blood flow supports nutrient absorption and barrier integrity.

### 5.2 The Microbiome‐Metabolome Interface: From Prebiotics to Pharmacologically Active Metabolites

Polyphenols selectively enrich commensal bacteria and suppress pathogens. Polyphenols increase *Bifidobacterium* and *Lactobacillus* while decreasing *Clostridia* and other pathogens, creating an anti‐inflammatory milieu (Sarubbo et al. 2023). These bacteria convert ellagitannins and anthocyanins into urolithins, which exert neuroprotective effects. Interestingly, decreased gut diversity and lower production of anti‐inflammatory metabolites such as urolithin A and SCFAs are associated with increased BBB permeability and brain aging (Láng et al. 2024). Diets rich in polyphenols, such as the Mediterranean diet, preserve gut diversity and cognitive function. Conversely, dysbiosis and increased production of p‐cresol and secondary bile acids can impair microglial function and cognition.

### 5.3 Immune and Cytokine Modulation

Polyphenols reduce pro‐inflammatory cytokines (IL‐6, TNF‐α) and increase anti‐inflammatory cytokines. Quercetin supplementation has been shown to reduce IL‐6 and TNF‐α and inhibit vascular calcification (Crescente and Moccia 2024). In the IBS synbiotic trial, reductions in IL‐8, TNF, and GM‐CSF corresponded with improved symptoms (Wierzbicka et al. 2025).

### 5.4 Metabolic and Endocrine Pathways

Polyphenols modulate glucose and lipid metabolism, partly by activating AMP‐activated protein kinase and inhibiting digestive enzymes. High‐phenolic EVOO improved lipid profiles and increased HDL (Kourek et al. 2025). Phenolic acids such as chlorogenic acid reduce glucose absorption and hepatic gluconeogenesis (Bassoli et al. 2008; Zuñiga et al. 2018). Improved metabolic control may indirectly benefit neuronal energy supply and reduce neuroinflammation.

### 5.5 Genetic Triangulation

Mendelian‐randomization (MR) studies offer insight into causality by using genetic variants as proxies for exposures. Researchers used genetic instruments for green‐tea intake and reported that higher genetically predicted tea intake was associated with slower progression to dementia in Parkinson's disease (odds ratio 0.87 per SD increase) (Li et al. 2022). Nominal associations were also observed with slower progression to depression, hyposmia, and insomnia. However, these findings should be interpreted as supportive evidence for causal consistency rather than definitive proof of causation. MR depends on several assumptions: the genetic instruments must be strongly associated with the exposure of interest, should not share confounding pathways with the outcome, and should not influence the outcome through horizontal pleiotropic routes outside the hypothesized exposure pathway. Additional concerns include weak‐instrument bias and NO Measurement Error (NOME) violations when exposure phenotypes are measured imprecisely, as well as limited cross‐population generalizability when instruments are derived largely from one ancestry or cultural context and applied more broadly (Burgess et al. 2024). For nutrition‐related traits such as tea intake, phenotype definition is particularly important because questionnaire‐based intake measures may imperfectly capture the biologically relevant exposure and may also tag correlated lifestyle patterns. Accordingly, future triangulation studies should pair MR with targeted metabolite measurements, sensitivity analyses for pleiotropy, replication across ancestries, and intervention trials that test whether genetically supported pathways can be engaged experimentally in humans.

### 5.6 Contradictions and Boundary Conditions

Although the pathway from microbial metabolites to barrier/inflammatory tone, vascular/endothelial biology, and neurobehavioral outcomes is biologically plausible, human evidence is heterogeneous, and intermediate biomarkers are often correlative rather than proven mediators; therefore, mechanistic claims in this review are framed as “associated with” or “consistent with” unless supported by randomized intervention effects or causal triangulation. For Memophenol (grape/blueberry extract) in mild cognitive impairment, improvements were observed in selected domains (e.g., processing speed and visuospatial learning), but most other cognitive and self‐report outcomes showed no statistically significant between‐group differences (Lopresti et al. 2023). In the wild blueberry RCT, vascular and some cognitive endpoints improved, yet no changes in cerebral blood flow or gut microbiota composition were detected, indicating that clinical effects (when present) do not necessarily require measurable shifts across every hypothesized intermediate step (Wood et al. 2023). Vascular/inflammatory trials also show boundary conditions: in OLIVAUS, no significant differences were observed between high‐ vs. low‐polyphenol EVOO treatments in the total sample, and antioxidant/inflammatory biomarker changes were more pronounced only in higher cardiometabolic‐risk strata (abdominal obesity or elevated hs‐CRP) (Sarapis et al. 2022). Inter‐individual microbiome capacity to generate bioactive metabolites is another plausible modifier: urolithin metabotypes vary after ellagitannin exposure and correlate with cardiometabolic risk, and in the MaPLE dietary crossover trial, urolithin metabotype B showed a larger zonulin decrease after a polyphenol‐rich diet than other metabotypes (Selma et al. 2018). Because baseline microbiome configurations can be stratified (including enterotype‐like clustering) and associated with habitual diet, future studies should combine baseline microbiome/metabotype characterization with targeted metabolite measurement and trials designed to test causal consistency, rather than assuming a single linear pathway applies across populations.

## 6 A Framework for Future Research: Biomarkers, Formulation, and Trial Design

Given the promising but heterogeneous evidence base summarized above, this section asks what study features are required to generate more robust, translatable, and mechanism‐resolved evidence. The focus shifts from describing current findings to specifying the biomarker, formulation, and design elements needed for the next generation of neuro–enteric polyphenol trials.

### 6.1 A Proposed Multi‐Omics Biomarker Panel for Neuro‐Enteric Trials

Integrating multi‐omics readouts into RCTs can clarify mechanisms and enable precision nutrition. Based on the reviewed evidence, we propose a core biomarker panel (Table 2) incorporating neurocognitive, vascular, immune, and microbiome/metabolome assessments.

**TABLE 2:** Proposed core biomarker panel. This table emphasizes that sampling windows should include baseline, acute (post‐prandial), and chronic time points to capture rapid vasodilatory effects and longer‐term adaptations. Stool samples should be collected at baseline and study end, while plasma and urine metabolites may require repeated sampling to capture fluctuations.

Domain	Example biomarkers and measures	Rationale
Neurocognitive	Comprehensive battery (RBANS or ENB‐2), executive function tests (Trail Making, Stroop), memory (Hopkins Verbal Learning), attention and processing speed; sleep quality (PSQI); mood (PHQ‐9, GAD‐7)	Detects domain‐specific cognitive changes; sleep and mood influence gut–brain signaling and may reflect polyphenol effects on neuromodulators (Uchida et al. 2024)
Vascular & endothelial	Flow‐mediated dilation (FMD), pulse wave velocity (PWV), blood pressure, cerebrovascular reactivity, carotid intima–media thickness, plasma nitric‐oxide metabolites	Polyphenols improve endothelial function and FMD; changes correlate with cognitive improvements (Wood et al. 2023)
Immune & inflammatory	High‐sensitivity C‐reactive protein (hs‐CRP), IL‐6, IL‐8, TNF‐α, GM‐CSF, endotoxin (LPS) levels; leukocyte gene expression	Reflects systemic and gut inflammation; reductions noted in IBS synbiotic trial (Wierzbicka et al. 2025)
Microbiome and metabolome	16S rRNA sequencing and metagenomics for *α*‐ and *β*‐diversity, relative abundance of *Bifidobacterium, Faecalibacterium*, and SCFA producers; targeted metabolomics for SCFAs (acetate, propionate, butyrate), phenyl‐γ‐valerolactones, urolithins, and catechin metabolites in plasma, urine, and cerebrospinal fluid	Captures microbial shifts and metabolite production that may mediate cognitive benefits (Sarubbo et al. 2023)
Metabolic	Fasting glucose, insulin, HOMA‐IR, lipid profile, lipoprotein (a), apolipoproteins, liver enzymes	Polyphenols improve lipid profiles and insulin sensitivity (Kourek et al. 2025)

Abbreviations: 16S *rRNA*, 16S ribosomal RNA, ENB 2, Esame Neuropsicologico Breve 2, FMD, flow‐mediated dilation, GAD‐7, Generalized Anxiety Disorder‐7, GM‐CSF, Granulocyte‐macrophage colony‐stimulating factor, HOMA‐IR, Homeostatic model assessment for insulin resistance, hs‐CRP, high‐sensitivity C‐reactive protein, IL‐6, Interleukin‐6, IL‐8, Interleukin‐8, LPS, Lipopolysaccharide, PHQ‐9, Patient Health Questionnaire‐9, PSQI, Pittsburgh Sleep Quality Index, PWV, pulse wave velocity, RBANS, Repeatable Battery for the Assessment of Neuropsychological Status. SCFAs, Short‐chain fatty acids, TNF‐α, Tumor necrosis factor alpha.

To advance the field from promising but heterogeneous signals to robust, translatable evidence, future research must adopt more rigorous and standardized methodologies. At the same time, feasibility considerations should be made explicit. Multi‐omics profiling can improve mechanistic resolution, but its implementation is constrained by assay cost, batch harmonization, biospecimen handling requirements, data‐integration complexity, and the need for bioinformatics pipelines that are reproducible across sites. Longitudinal neuro–enteric trials also might face practical challenges in sustaining dietary adherence, supplement fidelity, stool and blood sampling compliance, participant retention, and follow‐up funding beyond the initial intervention window. These issues are especially relevant when repeated targeted metabolomics, microbiome sequencing, vascular testing, and neurocognitive batteries are combined in one protocol. Future programs will therefore need staged biomarker panels, pre‐specified analytic plans, shared quality‐control frameworks, and realistic budgeting so that mechanistic ambition is matched by operational feasibility.

### 6.2 Formulation Science, Bioavailability, and Food Matrix Considerations

The efficacy of polyphenol interventions depends on dosage, formulation, food matrix, and co‐nutrients. High‐molecular‐weight proanthocyanidins have limited absorption; flavonoid glycosides require deglycosylation by microbiota before absorption (Tao et al. 2019). Lipid co‐ingestion enhances absorption of lipophilic polyphenols such as curcumin and resveratrol (Coradini et al. 2014; Zhou et al. 2021). Studies suggest that proteins can form complexes with polyphenols, increasing or decreasing bioavailability depending on the protein type and binding affinity (Ciupei et al. 2024). For example, milk proteins may reduce catechin bioavailability in tea, whereas co‐ingestion with olive oil enhances hydroxytyrosol absorption. Sustained intake is important because polyphenol metabolites may accumulate over weeks; the Memophenol and Cognigrape trials observed larger cognitive gains after 6–12 weeks than after 2 weeks (Amone et al. 2024). Processing methods (freeze‐drying, encapsulation) influence stability and palatability; matcha and blueberry powders provide convenient, whole‐food matrices that include fiber and micronutrients, which may synergize with polyphenols.

Neutral or negative trials, such as the cocoa‐extract arm of COSMOS, highlight the importance of using sufficiently high doses (≥ 500 mg/day flavanols) and ensuring formulation stability. Cocoa supplements may lose active epicatechins during manufacturing or storage. Additionally, baseline nutrient status matters—multivitamin co‐supplementation improved cognition, suggesting that addressing micronutrient deficiencies can potentiate polyphenol effects (Baker et al. 2023).

### 6.3 Trial Design Priorities for Next‐Generation Studies

**FIGURE 2:**
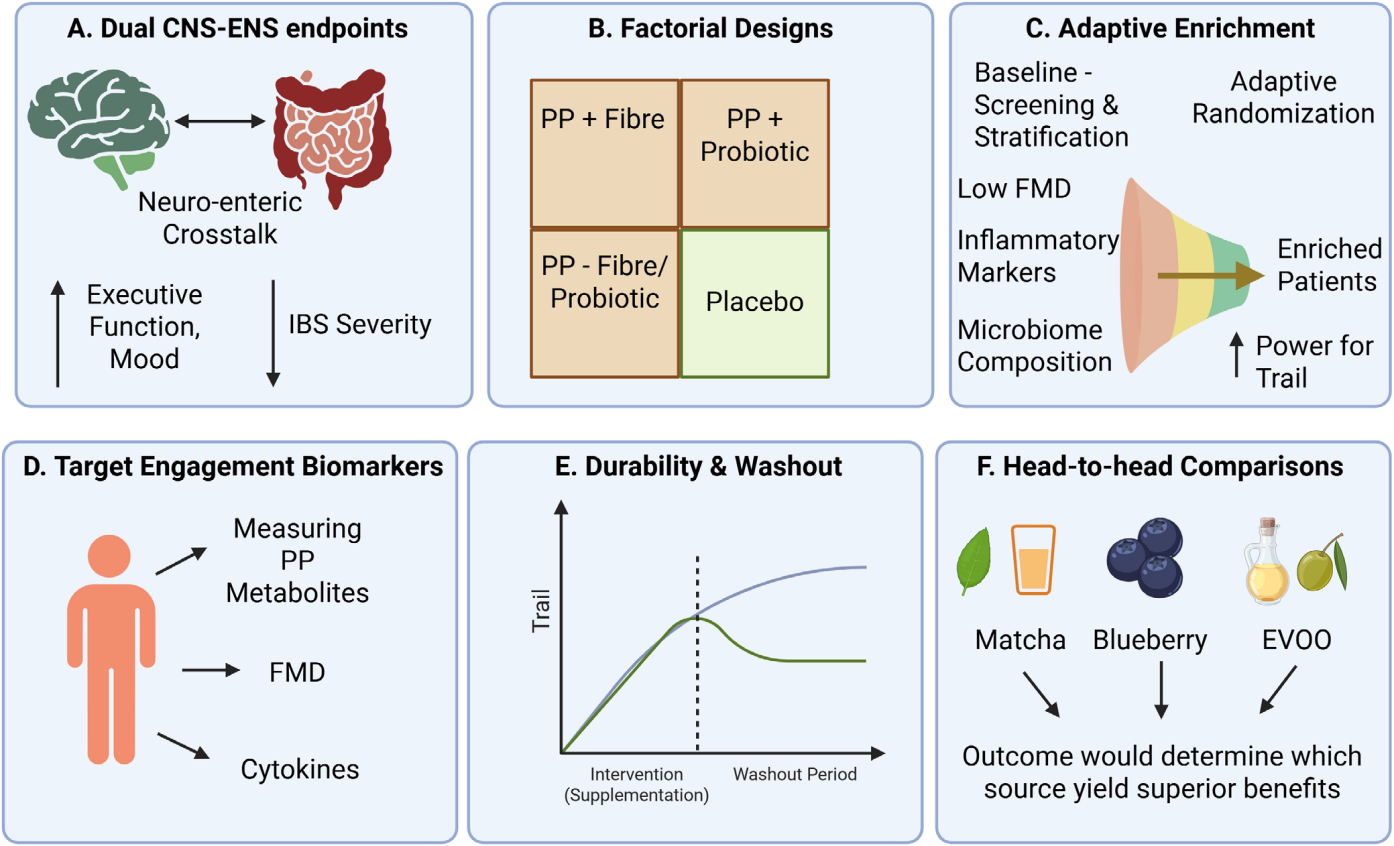
Trial design elements. From left to right: (A) Dual gut–brain endpoints, (B) factorial treatment assignment, (C) adaptive randomization based on baseline biomarkers, (D) target engagement markers, (E) washout period, (F) direct comparison of different sources.

To move beyond associative signals, future RCTs should incorporate mechanism‐anchored designs illustrated in Figure 2. (a) Dual CNS–ENS endpoints: Trials should include both cognitive and gastrointestinal outcomes. For example, evaluating the effect of berry polyphenols plus fiber on FMD, executive function, and IBS severity can reveal cross‐system benefits. (b) Factorial designs: Randomized factorial designs can disentangle the contributions of polyphenols and co‐nutrients (e.g., polyphenol ± probiotic/fiber). Such designs were fruitful in COSMOS (cocoa extract × multivitamin) and could be applied to berry + probiotic combinations. (c) Adaptive enrichment: Baseline vascular dysfunction, inflammation, or microbiome composition may modify responses. Adaptive randomization could enrich individuals with low FMD or high inflammatory markers to increase power. Genetic polymorphisms affecting polyphenol metabolism (e.g., COMT, gut microbial enterotypes) could be used as stratification factors. (d) Target engagement biomarkers: Trials should incorporate the biomarker panel described in section 6.1. Measuring plasma and urine polyphenol metabolites, FMD, and cytokines can confirm that interventions achieve biological effects. Use of wearable devices (sleep trackers, cognitive apps) could provide continuous data on mood and cognition. (e) Durability and washout: Many benefits were observed while supplementation continued; few trials assessed whether effects persist after discontinuation. Future studies should include washout periods to evaluate durability and possible rebound effects. (f) Head‐to‐head comparisons: Few studies directly compare different polyphenol sources. Comparative trials (e.g., matcha vs. blueberry vs. EVOO) could determine whether certain sources or combinations yield superior neuro–enteric benefits.

## 7 Translation and Public Health Implications

This section considers how the preceding mechanistic and clinical evidence might be translated into realistic dietary, regulatory, and public‐health strategies. It also highlights that feasibility, access, and implementation constraints will shape whether promising trial findings can be translated beyond tightly controlled research settings. While clinical trials often rely on concentrated extracts to achieve measurable effects within a study's timeframe, the goal is to inform public health guidance. The evidence strongly supports a food‐first strategy. Encouraging the regular consumption of a variety of polyphenol‐rich whole foods—such as a daily serving of berries, 2–3 cups of green tea, and the use of high‐quality EVOO—within the context of a balanced, plant‐predominant dietary pattern is a safe and effective approach to support gut and brain health.

From a regulatory perspective, there is a need for greater transparency and stronger evidence of standards for health claims on polyphenol supplements. Labels should accurately report the content of specific bioactive compounds and be supported by high‐quality clinical trial data. Finally, issues of equity and access must be addressed. Polyphenol‐rich fresh fruits and vegetables can be expensive and inaccessible to some populations. Public health policies that subsidize these foods, support community gardens, and provide culinary education can help ensure that the benefits of a polyphenol‐rich diet are accessible to all.

## 8 Conclusion

The body of evidence linking dietary polyphenols to improved outcomes in non‐communicable chronic diseases is growing in strength and sophistication. Recent randomized trials of berries, grapes, matcha, and high‐phenolic olive oil provide consistent, albeit modest, evidence for benefits in memory, executive function, vascular reactivity, and enteric health. These clinical signals are supported by plausible biological mechanisms, with improved endothelial function and prebiotic modulation of the gut microbiome emerging as central pillars of polyphenol bioactivity. Genetic analyses further strengthen the case for a causal neuroprotective role for certain polyphenols. However, the field is at a critical juncture. The heterogeneity of interventions, small effect sizes, and inconsistent use of biomarkers remain significant barriers to clinical translation. The path forward requires a concerted shift toward more rigorous, mechanism‐oriented research. The adoption of a standardized multi‐omics biomarker panel, coupled with innovative trial designs that integrate CNS and ENS endpoints and enrich responder populations, will be essential. By embracing these strategies, the scientific community can move closer to harnessing the full potential of dietary polyphenols to develop effective, personalized nutrition strategies that mitigate the rising burden of NCCDs and promote healthy aging across the lifespan.

## Author Contributions

**Jannatul Wahid Munami:** visualization, investigation. **Nusrat Jahan Shawon:** conceptualization, writing – review and editing, formal analysis, supervision, project administration, resources. **Rajib Das:** visualization, formal analysis. **Adnan Akif:** writing – original draft, investigation, conceptualization, software, data curation.

## Data Availability

Data sharing not applicable to this article as no datasets were generated or analysed during the current study.

## References

[fsn371856-bib-0001] Alia, S. , A. Di Paolo , V. Membrino , T. Di Crescenzo , and A. Vignini . 2024. “Beneficial Effects on Oxidative Stress and Human Health by Dietary Polyphenols.” Antioxidants 13, no. 11: 1314. 10.3390/antiox13111314. 39594456 PMC11591040

[fsn371856-bib-0002] Amone, F. , A. Spina , A. Perri , et al. 2024. “Standardized Grape ( *Vitis vinifera* L.) Extract Improves Short‐ and Long‐Term Cognitive Performances in Healthy Older Adults: A Randomized, Double‐Blind, and Placebo‐Controlled Trial.” Food 13, no. 18: 2999. 10.3390/foods13182999. PMC11431441 39335927

[fsn371856-bib-0003] Baker, L. D. , J. E. Manson , S. R. Rapp , et al. 2023. “Effects of Cocoa Extract and a Multivitamin on Cognitive Function: A Randomized Clinical Trial.” Alzheimer's & Dementia: The Journal of the Alzheimer's Association 19, no. 4: 1308–1319. 10.1002/alz.12767. PMC10011015 36102337

[fsn371856-bib-0004] Barona, J. , J. C. Aristizabal , C. N. Blesso , J. S. Volek , and M. L. Fernandez . 2012. “Grape Polyphenols Reduce Blood Pressure and Increase Flow‐Mediated Vasodilation in Men With Metabolic Syndrome1.” Journal of Nutrition 142, no. 9: 1626–1632. 10.3945/jn.112.162743. 22810991

[fsn371856-bib-0005] Bassoli, B. K. , P. Cassolla , G. R. Borba‐Murad , et al. 2008. “Chlorogenic Acid Reduces the Plasma Glucose Peak in the Oral Glucose Tolerance Test: Effects on Hepatic Glucose Release and Glycaemia.” Cell Biochemistry and Function 26, no. 3: 320–328. 10.1002/cbf.1444. 17990295

[fsn371856-bib-0006] Brâkenhielm, E. , R. Cao , and Y. Cao . 2001. “Suppression of Angiogenesis, Tumor Growth, and Wound Healing by Resveratrol, a Natural Compound in Red Wine and Grapes.” FASEB Journal 15, no. 10: 1798–1800. 10.1096/fj.01-0028fje. 11481234

[fsn371856-bib-0007] Burgess, S. , B. Woolf , A. M. Mason , M. Ala‐Korpela , and D. Gill . 2024. “Addressing the Credibility Crisis in Mendelian Randomization.” BMC Medicine 22, no. 1: 374. 10.1186/s12916-024-03607-5. 39256834 PMC11389083

[fsn371856-bib-0008] Ciupei, D. , A. Colişar , L. Leopold , A. Stănilă , and Z. M. Diaconeasa . 2024. “Polyphenols: From Classification to Therapeutic Potential and Bioavailability.” Food 13, no. 24: 4131. 10.3390/foods13244131. PMC11675957 39767073

[fsn371856-bib-0009] Coradini, K. , F. O. Lima , C. M. Oliveira , et al. 2014. “Co‐Encapsulation of Resveratrol and Curcumin in Lipid‐Core Nanocapsules Improves Their In Vitro Antioxidant Effects.” European Journal of Pharmaceutics and Biopharmaceutics 88, no. 1: 178–185. 10.1016/j.ejpb.2014.04.009. 24780440

[fsn371856-bib-0010] Crescente, G. , and S. Moccia . 2024. “Editorial: Carotenoids, Polyphenols and Phytocannabinoids: New Perspectives in the Prevention of Chronic Diseases.” Frontiers in Pharmacology 15: 1419129. 10.3389/fphar.2024.1419129. 38808255 PMC11130484

[fsn371856-bib-0011] Domínguez‐López, I. , A. López‐Yerena , A. Vallverdú‐Queralt , M. Pallàs , R. M. Lamuela‐Raventós , and M. Pérez . 2025. “From the Gut to the Brain: The Long Journey of Phenolic Compounds With Neurocognitive Effects.” Nutrition Reviews 83, no. 2: e533–e546. 10.1093/nutrit/nuae034. 38687609 PMC11723161

[fsn371856-bib-0012] Duda‐Chodak, A. , T. Tarko , P. Satora , and P. Sroka . 2015. “Interaction of Dietary Compounds, Especially Polyphenols, With the Intestinal Microbiota: A Review.” European Journal of Nutrition 54, no. 3: 325–341. 10.1007/s00394-015-0852-y. 25672526 PMC4365176

[fsn371856-bib-0013] Gerstenmeyer, E. , S. Reimer , E. Berghofer , H. Schwartz , and G. Sontag . 2013. “Effect of Thermal Heating on Some Lignans in Flax Seeds, Sesame Seeds and Rye.” Food Chemistry 138, no. 2–3: 1847–1855. 10.1016/j.foodchem.2012.11.117. 23411317

[fsn371856-bib-0014] Hollman, P. C. H. 2004. “Absorption, Bioavailability, and Metabolism of Flavonoids.” Pharmaceutical Biology 42, no. sup1: 74–83. 10.3109/13880200490893492.

[fsn371856-bib-0015] Kourek, C. , E. Makaris , P. Magiatis , et al. 2025. “Effects of High‐Phenolic Extra Virgin Olive Oil (EVOO) on the Lipid Profile of Patients With Hyperlipidemia: A Randomized Clinical Trial.” Nutrients 17, no. 15: 2543. 10.3390/nu17152543. 40806126 PMC12348208

[fsn371856-bib-0016] Landete, J. M. 2011. “Ellagitannins, Ellagic Acid and Their Derived Metabolites: A Review About Source, Metabolism, Functions and Health.” Food Research International 44, no. 5: 1150–1160. 10.1016/j.foodres.2011.04.027.

[fsn371856-bib-0017] Láng, L. , S. McArthur , A. S. Lazar , et al. 2024. “Dietary (Poly) Phenols and the Gut–Brain Axis in Ageing.” Nutrients 16, no. 10: 1500. 10.3390/nu16101500. 38794738 PMC11124177

[fsn371856-bib-0018] Leikert, J. F. , T. R. Räthel , P. Wohlfart , V. Cheynier , A. M. Vollmar , and V. M. Dirsch . 2002. “Red Wine Polyphenols Enhance Endothelial Nitric Oxide Synthase Expression and Subsequent Nitric Oxide Release From Endothelial Cells.” Circulation 106, no. 13: 1614–1617. 10.1161/01.cir.0000034445.31543.43. 12270851

[fsn371856-bib-0019] Li, C. , J. Lin , T. Yang , and H. Shang . 2022. “Green Tea Intake and Parkinson's Disease Progression: A Mendelian Randomization Study.” Frontiers in Nutrition 9: 848223. 10.3389/fnut.2022.848223. 35719152 PMC9199515

[fsn371856-bib-0020] Lin, K. , Y. Li , E. D. Toit , L. Wendt , and J. Sun . 2021. “Effects of Polyphenol Supplementations on Improving Depression, Anxiety, and Quality of Life in Patients With Depression.” Frontiers in Psychiatry 12: 765485. 10.3389/fpsyt.2021.765485. 34819888 PMC8606635

[fsn371856-bib-0021] Lopresti, A. L. , S. J. Smith , C. Pouchieu , et al. 2023. “Effects of a Polyphenol‐Rich Grape and Blueberry Extract (Memophenol) on Cognitive Function in Older Adults With Mild Cognitive Impairment: A Randomized, Double‐Blind, Placebo‐Controlled Study.” Frontiers in Psychology 14: 1144231. 10.3389/fpsyg.2023.1144231. 37063535 PMC10095830

[fsn371856-bib-0022] Mattila, P. , J. Hellström , and R. Törrönen . 2006. “Phenolic Acids in Berries, Fruits, and Beverages.” Journal of Agricultural and Food Chemistry 54, no. 19: 7193–7199. 10.1021/jf0615247. 16968082

[fsn371856-bib-0023] Merra, G. , A. Noce , G. Marrone , et al. 2021. “Influence of Mediterranean Diet on Human Gut Microbiota.” Nutrients 13, no. 1: 7. 10.3390/nu13010007. PMC7822000 33375042

[fsn371856-bib-0024] Nediani, C. , J. Ruzzolini , and M. Dinu . 2024. “Oxidative Stress and Inflammation as Targets for Novel Preventive and Therapeutic Approaches in Non‐Communicable Diseases III.” Antioxidants 13, no. 11: 1404. 10.3390/antiox13111404. 39594546 PMC11591297

[fsn371856-bib-0025] Qiu, X. , M. Zhang , X. Yang , N. Hong , and C. Yu . 2013. “ *Faecalibacterium prausnitzii* Upregulates Regulatory T Cells and Anti‐Inflammatory Cytokines in Treating TNBS‐Induced Colitis.” Journal of Crohn's and Colitis 7, no. 11: e558–e568. 10.1016/j.crohns.2013.04.002. 23643066

[fsn371856-bib-0026] Renard, C. M. G. C. , A. A. Watrelot , and C. Le Bourvellec . 2017. “Interactions Between Polyphenols and Polysaccharides: Mechanisms and Consequences in Food Processing and Digestion.” Trends in Food Science & Technology, Special Issues From the 29th EFFoST International Conference 60: 43–51. 10.1016/j.tifs.2016.10.022.

[fsn371856-bib-0027] Rodríguez Galdón, B. , E. m. Rodríguez Rodríguez , and C. Díaz Romero . 2008. “Flavonoids in Onion Cultivars ( *Allium cepa* L.).” Journal of Food Science 73, no. 8: C599–C605. 10.1111/j.1750-3841.2008.00903.x. 19019103

[fsn371856-bib-0028] Rodríguez‐Daza, M. C. , E. C. Pulido‐Mateos , J. Lupien‐Meilleur , D. Guyonnet , Y. Desjardins , and D. Roy . 2021. “Polyphenol‐Mediated Gut Microbiota Modulation: Toward Prebiotics and Further.” Frontiers in Nutrition 8: 1–24. 10.3389/fnut.2021.689456. PMC8276758 34268328

[fsn371856-bib-0029] Sarapis, K. , E. S. George , W. Marx , et al. 2022. “Extra Virgin Olive Oil High in Polyphenols Improves Antioxidant Status in Adults: A Double‐Blind, Randomized, Controlled, Cross‐Over Study (OLIVAUS).” European Journal of Nutrition 61, no. 2: 1073–1086. 10.1007/s00394-021-02712-y. 34716791

[fsn371856-bib-0030] Sarubbo, F. , D. Moranta , S. Tejada , M. Jiménez , and S. Esteban . 2023. “Impact of Gut Microbiota in Brain Ageing: Polyphenols as Beneficial Modulators.” Antioxidants 12, no. 4: 812. 10.3390/antiox12040812. 37107187 PMC10134998

[fsn371856-bib-0031] Selma, M. V. , A. González‐Sarrías , J. Salas‐Salvadó , et al. 2018. “The Gut Microbiota Metabolism of Pomegranate or Walnut Ellagitannins Yields Two Urolithin‐Metabotypes That Correlate With Cardiometabolic Risk Biomarkers: Comparison Between Normoweight, Overweight‐Obesity and Metabolic Syndrome.” Clinical Nutrition 37, no. 3: 897–905. 10.1016/j.clnu.2017.03.012. 28347564

[fsn371856-bib-0032] Singh, B. N. , S. Shankar , and R. K. Srivastava . 2011. “Green Tea Catechin, Epigallocatechin‐3‐Gallate (EGCG): Mechanisms, Perspectives and Clinical Applications.” Biochemical Pharmacology 82, no. 12: 1807–1821. 10.1016/j.bcp.2011.07.093. 21827739 PMC4082721

[fsn371856-bib-0033] Small, G. W. , P. Siddarth , Z. Li , et al. 2018. “Memory and Brain Amyloid and Tau Effects of a Bioavailable Form of Curcumin in Non‐Demented Adults: A Double‐Blind, Placebo‐Controlled 18‐Month Trial.” American Journal of Geriatric Psychiatry: Official Journal of the American Association for Geriatric Psychiatry 26, no. 3: 266–277. 10.1016/j.jagp.2017.10.010. 29246725

[fsn371856-bib-0034] Tao, W. , Y. Zhang , X. Shen , et al. 2019. “Rethinking the Mechanism of the Health Benefits of Proanthocyanidins: Absorption, Metabolism, and Interaction With Gut Microbiota.” Comprehensive Reviews in Food Science and Food Safety 18, no. 4: 971–985. 10.1111/1541-4337.12444. 33336996

[fsn371856-bib-0035] Uchida, K. , K. Meno , T. Korenaga , et al. 2024. “Effect of Matcha Green Tea on Cognitive Functions and Sleep Quality in Older Adults With Cognitive Decline: A Randomized Controlled Study Over 12 Months.” PLoS One 19, no. 8: e0309287. 10.1371/journal.pone.0309287. 39213264 PMC11364242

[fsn371856-bib-0036] Vyas, C. M. , J. E. Manson , H. D. Sesso , et al. 2024. “Effect of Multivitamin‐Mineral Supplementation Versus Placebo on Cognitive Function: Results From the Clinic Subcohort of the COcoa Supplement and Multivitamin Outcomes Study (COSMOS) Randomized Clinical Trial and Meta‐Analysis of 3 Cognitive Studies Within COSMOS.” American Journal of Clinical Nutrition 119, no. 3: 692–701. 10.1016/j.ajcnut.2023.12.011. 38244989 PMC11103094

[fsn371856-bib-0037] Wierzbicka, A. , B. Khaidakov , O. Zakerska‐Banaszak , et al. 2025. “Effects of a Polyphenol‐Rich Extract Blend, Probiotics, and Hydrolyzed Fiber on Quality of Life and Gut Health Markers in Patients With Irritable Bowel Syndrome‐A Randomized, Double‐Blind, Placebo‐Controlled Trial.” Frontiers in Nutrition 12: 1603011. 10.3389/fnut.2025.1603011. 40693198 PMC12277158

[fsn371856-bib-0038] Wood, E. , S. Hein , R. Mesnage , et al. 2023. “Wild Blueberry (Poly) Phenols Can Improve Vascular Function and Cognitive Performance in Healthy Older Individuals: A Double‐Blind Randomized Controlled Trial.” American Journal of Clinical Nutrition 117, no. 6: 1306–1319. 10.1016/j.ajcnut.2023.03.017. 36972800 PMC10315404

[fsn371856-bib-0039] Zhou, H. , B. Zheng , and D. J. McClements . 2021. “In Vitro Gastrointestinal Stability of Lipophilic Polyphenols Is Dependent on Their Oil‐Water Partitioning in Emulsions: Studies on Curcumin, Resveratrol, and Quercetin.” Journal of Agricultural and Food Chemistry 69, no. 11: 3340–3350. 10.1021/acs.jafc.0c07578. 33689331

[fsn371856-bib-0040] Zuñiga, L. Y. , M. C. A. Aceves‐de la Mora , M. González‐Ortiz , J. L. Ramos‐Núñez , and E. Martínez‐Abundis . 2018. “Effect of Chlorogenic Acid Administration on Glycemic Control, Insulin Secretion, and Insulin Sensitivity in Patients With Impaired Glucose Tolerance.” Journal of Medicinal Food 21, no. 5: 469–473. 10.1089/jmf.2017.0110. 29261010

